# Synthesis of irregular graphene oxide tubes using green chemistry and their potential use as reinforcement materials for biomedical applications

**DOI:** 10.1371/journal.pone.0185235

**Published:** 2017-09-21

**Authors:** Ángel Serrano-Aroca, Sanjukta Deb

**Affiliations:** 1 Bioengineering & Cellular Therapy Group, Facultad de Veterinaria y Ciencias Aplicadas, Universidad Católica de Valencia “San Vicente Mártir”, C/Guillem de Castro 94,Valencia, Spain; 2 Division of Tissue Engineering & Biophotonics, King’s College London, Dental Institute, London, Tower Wing, Guy’s Hospital, London, United Kingdom; Institute of Materials Science, GERMANY

## Abstract

Micrometer length tubes of graphene oxide (GO) with irregular form were synthesised following facile and green metal complexation reactions. These materials were obtained by crosslinking of GO with calcium, zinc or strontium chlorides at three different temperatures (24, 34 and 55°C) using distilled water as solvent for the compounds and following a remarkably simple and low-cost synthetic method, which employs no hazardous substances and is conducted without consumption of thermal or sonic energy. These irregular continuous GO networks showed a very particular interconnected structure by Field Emission Scanning Electron Microscopy with Energy-Disperse X-Ray Spectroscopy for elemental analysis and High-resolution Transmission Electron Microscopy with Scanning Transmission Electron Microscope Dark Field Imaging, and were analysed by Raman Spectroscopy. To demonstrate the potential use of these 3D GO networks as reinforcement materials for biomedical applications, two composites of calcium alginate with irregular tubes of GO and with single GO nanosheets were prepared with the same amount of GO and divalent atoms and analysed. Thus, the dynamic-mechanical modulus of the composites synthesised with the 3D crosslinked GO networks showed a very significant mechanical improvement due to marked microstructural changes confirmed by confocal microscopy, differential scanning calorimetry and Fourier transform infrared spectroscopy.

## Introduction

Scientific and technological advances continue to successfully exploit the fascinating properties of graphene, a class of materials with a two dimensional structure that possess an array of unique properties. It consists of single atom thick monolayers hexagonally arranged sp^2^ bonded carbon atoms forming a lattice, the thinnest known substance made from carbon, with a large specific surface area that is exceptionally strong and stiff[1] with a high thermal[2] and electrical[3] conductivity. This two-dimensional sp^2^-hybridized carbon material itself is inert to many reactants[4] and all graphene-bound anions reported to date possess major limitations in terms of their electronic tunability and coordination chemistry[5]. However, graphene oxide (GO), a highly defective form of graphene with a disrupted sp^2^-bonding network, offers imperfect polar surfaces and secondary reactive sites capable of covalent and non-covalent chemical modification with organic and inorganic molecules [5]. Research on graphene oxide specifically exploiting the high oxygen containing functional groups has seen new frontiers with promising applications as nanomaterials for analytical chemistry, heavy metal pollutant cleaning up agents[6–10], biotechnology[11], electronics, optics, energy storage, bio and chemical sensors and electronic nano-devices[12–14]. Tubular carbon nanomaterials such as carbon nanotubes (CNTs), discovered by Iijima[15], have also been in the research forefront in the last decade due to their excellent electrical properties with a superb conductivity, remarkable mechanical strength and modulus with many potential technological applications [16]. However, newer materials such as Aerographite with micro and nano tubular network structures[17] have been reported to exhibit very high surface area and large free volume with low density that are flexible and possess outstanding mechanical properties with enormous prospect in supercapacitors to electrocatalyst applications [18,19]. Aerographite decorated by ZnO nanocrystallites have been reported that have promising applications in optoelectronic technologies[20], aerographite-GaN hybrid networks as possible next generation materials for electronic, photonic and sensors applications[21], and carbon nanowall (CNW) tetrapods coupled to nanocrystalline diamond in a 3D hybrid form as an excellent candidate for energy storage[22] are other remarkable examples of advanced 3D carbon-based materials. Also very recently, introduction of ionic groups in the graphene lattice have been reported, exemplifying the first of an ionic graphene salt containing boron[23]. These new materials consist of single functionalized graphene or GO sheets, which adhere together by van der Waals forces with residual π-π stacking between the faces of each sheet. 3D graphene oxide networks can also be synthesised by metal ion coordination chemistry, which has been shown to be an effective way of interconnecting or cross-linking carbon nanotubes (CNTs) [24], GO [25,26], or chemically modified graphene (CMG) with CNTs[27] through the oxygen-containing functional groups via divalent cations (Ca^2+^, Mg^2+^, Cu^2+^ and Pb^2+^).

GO can also be readily manipulated via chemical functionalization and has been utilized to develop highly conductive nanocomposite of polystyrene with uniformly dispersed graphene sheets[28]. The hydrophilic functional groups present on the edges and basal planes of graphene oxide allow them to be readily exfoliated into monolayer sheets to yield stable suspensions in water. Since GO offers different wettability it enables interaction with biological molecules such as DNA, proteins via adsorption and or chemical bonding, which has prompted several biomedical applications. Various applications such as drug/gene delivery[29], reinforcing fillers in ternary composites to enhance properties of drug delivery carriers, photodyanamic therapy, functionalisation of GO for anti-cancer therapy[30,31] and for siRNA delivery into neural stem cells to enhance neuronal differentiation have been reported. [32]. Furthermore, the sharp irregular edges present in GO are being exploited for antibacterial activity and recent reports show that they are effective towards Gram positive bacteria [33]. Graphene oxide inclusion in orthopaedic bone cements based on poly(methyl methacrylate) have also been reported to enhance mechanical properties, particularly the fracture toughness and fatigue properties[34] and as additives or coatings leading to the next generation of metals for biomedical applications [35]. Notably, graphene oxide is being explored in bone, cardiac, cartilage and neural tissue engineering applications. Composite formulations with poly(vinyl alcohol-chitosan and GO nanofibers were reported to enhance the mechanical properties with excellent biocompatibility and were deemed feasible as artificial cartilage [36], whilst a layer by layer assembly of cells within GO sheets were built to create a multi-layer functional tissue construct from cardiomyocytes and endothelial cells[37].

The promising properties of GO with its flat two dimensional structure and the availability of functional groups with the potential of gelation and strong covalent interactions led us to explored the formation of 3D GO networks synthesised via crosslinking, using three different cations (Ca ^2+^, Zn^2+^ and Sr^2+^) at three different temperatures to expand the repertoire in biomedical applications. This approach will enable the inclusion of zinc and strontium in the matrix networks, which impart antibacterial properties[38,39] to biomedical composites especially where surface contamination is a potential hazard. Additionally the presence of ions such as strontium and zinc are also known to accelerate bone healing and possess antibacterial properties, thus the incorporation of these ionic species within scaffolds for bone regeneration is of significance.

Alginate is considered a very promising polysaccharide polymer that can be isolated from renewable sources such as brown algae[40] or microorganisms[41]. with applications in water treatment[42], an efficient alternative source for non-biodegradable plastic packaging materials[43] and has many potential biotechnological applications[44,45] since it is nontoxic, biocompatible, bioerodible and has a relatively low-cost in comparison with other polymeric materials. Sodium alginate can form hydrogels in the presence of divalent cations such as Ca^2+^ due to ionic cross-linking, owing to their chelation with the carboxyl groups[46]. Crosslinked alginate chains form a buckled structure in the shape of an “egg box” whose cavity can accommodate divalent cations in the gelation process of water soluble sodium alginate[47]. However, alginates possess poor mechanical properties, which restrict their widespread use in many biomedical and industrial applications. Hence, reinforcement of alginates via the incorporation of graphene oxide (GO)[48,49] is promising with significant improvements reported using films of sodium alginate [50] and fibers of calcium alginate [51]. More recently, GO-alginate composite hydrogels synthesised by the direct mixing method [52] using high amounts of calcium chloride have exhibited great enhancement of compression performance and water diffusion with the addition of even a minuscule amount of graphene oxide[53]. This improvement of water diffusion is also very desirable in many biomedical applications due to mass transport plays an important role in cell survival [54].

In this study, we hypothesize that using a facile and green route to create graphene oxides crosslinked with divalent cations such as calcium, zinc or strontium will yield three-dimensional GO networks that can be safely used for biomedical applications by virtue of the synthetic route and enable composite formulation with enhanced mechanical properties, which is exemplified through forming GO/calcium alginate composites in comparison to the use of single GO nanosheets and consequently also provide a simple process to include cations that enhance antibacterial effects and in trace amounts facilitate bone healing, thus can be explored for scaffolds for bone and wound healing applications.

## Materials and methods

### Materials

Calcium chloride (≥ 93.0%, Sigma-Aldrich), Zinc chloride (≥ 98.0%, Sigma-Aldrich), Strontium chloride(≥ 99.99%, Sigma-Aldrich) as crosslinkers, graphene oxide (Sigma-Aldrich) and sodium alginate (Sigma-Aldrich) were used without further purification.

### Synthesis

Graphene oxide was dispersed in distilled water and mixed with 10% (w/v in g/mL) calcium chloride with continuous stirring for 2 hours at 24, 34 and 55°C to obtain the GO networks coordinated by divalent calcium cations. The complex compounds synthesised were purified by centrifugation and washing twice with distilled water. The same reaction was performed using zinc chloride and strontium chloride to produce similar divalent complex compounds and these coordination compounds of crosslinked GO are hereafter referred to as cGO. The graphene oxide complexation compounds were subsequently vacuum dried at 60°C for 48 hours. This synthetic procedure was assessed by Raman spectroscopy and showed excellent stability and repeatability.

The reinforced calcium alginate composites were synthesised by complexation reaction of calcium chloride (6 wt.%) and GO (1 wt. %) both dissolved in water under continuous stirring over 2 hours at 24°C, and subsequently mixing with a 2% (w/v in g/mL) aqueous solution of sodium alginate. The amount of each component were calculated to yield a composite composed of 6 wt.% of calcium chloride and 1 wt.% of GO both percentages are with respect to the mass of sodium alginate used in the preparation. Calcium alginate composites were also prepared with the same amount of graphene oxide and calcium cations but without following the complexation reaction to enable comparison. These composites are referred as cGO/Alg and GO/Alg respectively.

### Methods

Raman Spectroscopy was performed on a confocal micro-Raman spectrometer (Renishaw inVia) with an argon ion laser at an excitation wavelength of 633 nm from 1000 to 3000 cm^-1^. All scans were taken with x20 lens at 600 l mm^-1^ grating. Samples for Raman analysis were prepared by deposition of dry samples onto a glass substrate. Field Emission Scanning Electron Microscopy (FESEM, Zeiss Ultra 55 Model) with Energy-Disperse X-Ray Spectroscopy was operated at an accelerating voltage of 2 kV to observe the morphology of GO and the crosslinked GO networks at a magnification of 50000, and at 20 kV for the elemental analysis. High-resolution Transmission Electron Microscopy (HR-TEM) with Scanning Transmission Electron Microscope (STEM) Dark Field Imaging at a magnification of 300000 was performed using a JEOL-TEM 2100F 200 kV electron microscope dispersing the samples with dichloromethane in an ultrasonic bath for ten minutes and subsequent drying at room temperature. The micrographs of the calcium alginate composites were taken on a TSM Tandem scanning confocal microscope (TSM) (Noran Instruments, Middleton, WI, USA) with a Motorized Lab Jack (Thorlabs, LTD.,Ely, UK) in conjunction with an EMCCD camera iXon 885 EM-CCD (electron multiplying charge-coupled device) with iQ capture software (Andor Technology, Northern Ireland, UK). A Jade series differential scanning calorimeter was used to determine the thermal properties and Perkin Elmer Jade series software to process raw data. 10–20mg samples were carefully placed and sealed in aluminium pans (Perkin Elmer). The scans were carried out with reference pan calibrated using Indium^49^ under a Nitrogen^7^ atmosphere. The vacuum dried samples were subjected to a cooling scan from room temperature down to -30°C, followed by a heating scan up to 300°C at 10°C/min. These DSC scans were performed twice for each sample to ensure their reproducibility. A Perkin-Elmer DMA-800 dynamic mechanical analyser at a frequency of l Hz was used to determine the temperature dependence of storage modulus (E'), loss modulus (E”), and loss tangent (tan δ) from -50°C to 90°C of the dry samples with a heating rate of 1 K/min. FTIR transmission spectroscopy with a range of 650 to 4000 cm^-1^ was carried on a Perkin Elmer Spectrum One Fourier Transforms Infrared spectrometer.

## Results and discussion

**Fig 1:**
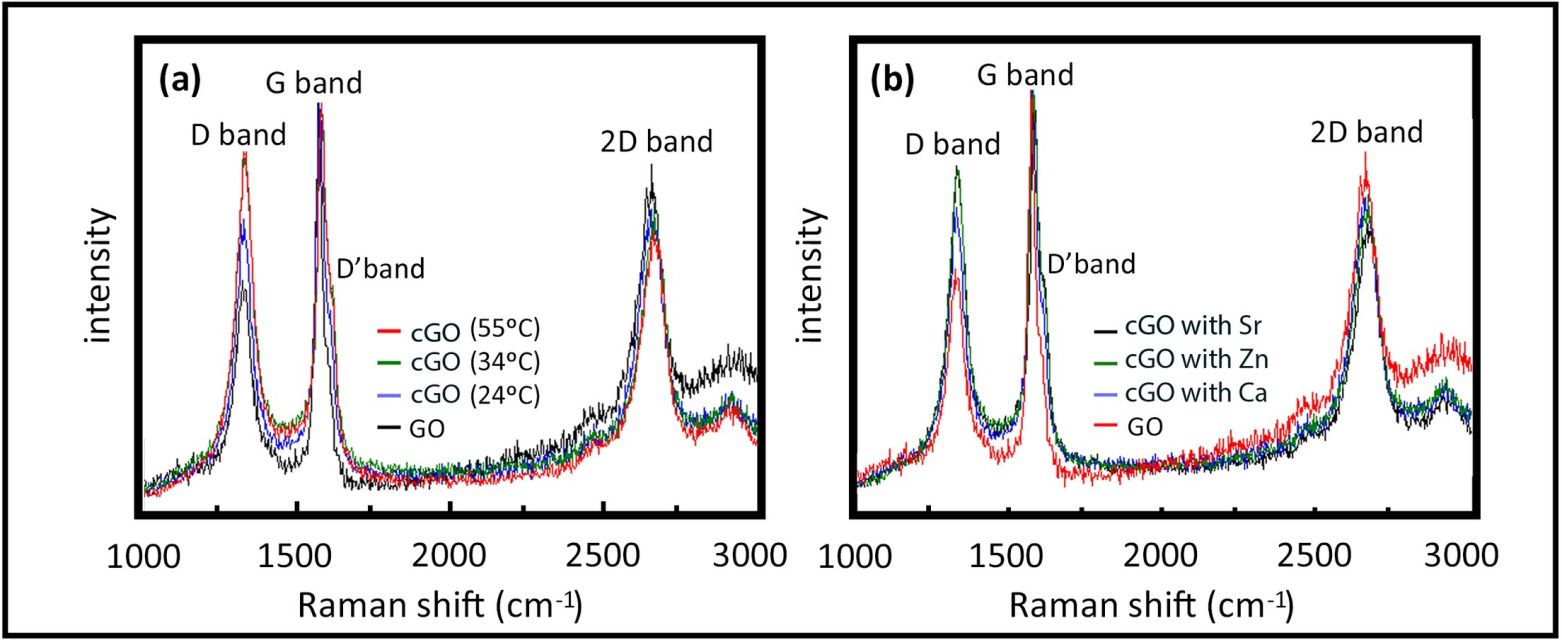
Raman spectroscopy. A comparison of the Raman spectra of (a) GO with cGO crosslinked with Ca at 24, 34 and 55°C, and (b) GO with cGO crosslinked with Ca, Zn and Sr at 24°C.

When divalent salts such as calcium chloride are dissociated in distilled water and agitated in an aqueous solution of GO, a complexation reaction occurs between the Ca^2+^ cations and the oxygen-containing functional groups, producing a continuous growing GO network due to the continuous bridging of the GO nanosheets by the divalent atoms tightly bonded by means of four oxygen atoms[25]. Raman spectroscopy is frequently used to determine defects and ordered/disordered structures of graphene. The D band is recognized to be a disordered band originating in structural defects, edge effects and dangling sp^2^ carbon bonds that break the symmetry. The intensity ratio of the D band to the G band (I_D_/I_G_) is generally accepted as representative of the defect/disordered carbon structure[55,56]. The effect of the reaction temperature and the type of divalent ions on the complexation reaction is shown in the Raman spectra (excitation with a 633 nm laser) in Fig 1A and 1B respectively. The GO complex networks synthesised show a very significant increase in the intensity of the D band with respect to that of GO. In addition, the I_D_/I_G_ ratios of GO and cGO when crosslinked with Zn^2+^, Sr^2+^ and Ca^2+^ synthesised at 24°C are 0.96, 1.31, 1.25 and 1.11 respectively whilst the temperature of the reaction also had an effect and using calcium as the chelating agent the values were 1.17 and 1.26 at 34 and 55°C respectively. This clearly indicate an increase, demonstrating a significant level of disorder at the carbon edges due to the increase in defects and loss of symmetry arising due to the random bridging of the divalent atoms in the GO network. The intensity of these D-bands also decreases dramatically with increasing number of graphene layers[57], which indicates that crosslinking at the edges also occurs producing a 2D growth of the GO nanosheets interlinked by the divalent atoms. Thus, Fig 1 shows how the D-band intensity increases dramatically after the formation of the graphene oxide complexation networks and increasing temperature from 24 to 34°C indicate that the number of graphene layers decrease. The increase of the I_D_/I_G_ ratios with increasing temperature, in spite of the broading of the G band due to the increase of the small D’ shoulder band, supports this last statement. This D’ shoulder appearing at around 1621 cm^-1^ in GO, related to the disorder of edge carbons[58], broadens on increasing the reaction temperature (Fig 1A) and when using different divalent salts (Fig 1B). This fact, once again, is in good agreement with the formation of complexation bonds at the edges. However, the D-band of Fig 1B shows that crosslinking of GO is much more effective using Zn^2+^ and Sr^2+^ at room temperature with I_D_/I_G_ ratios, as mentioned, close to that obtained with Ca^2+^ at 55°C.

**Fig 2:**
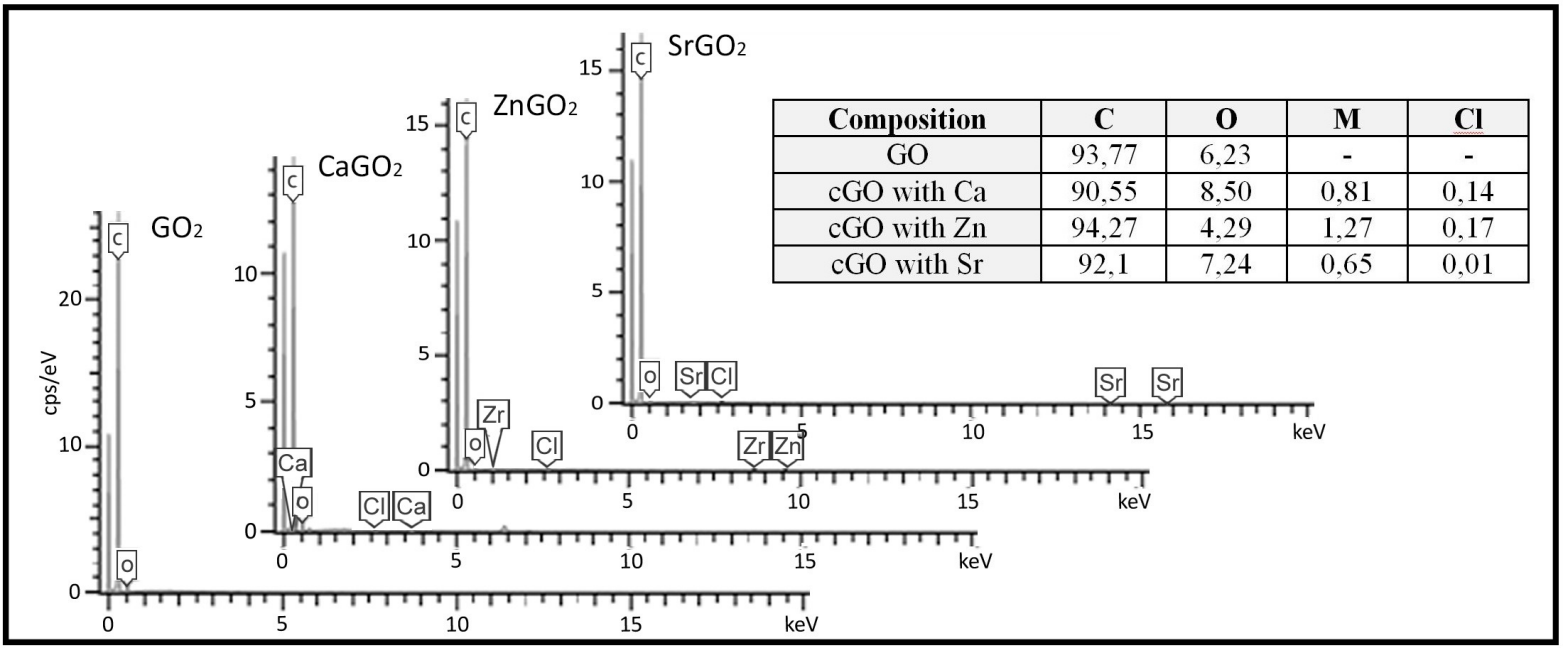
EDS analysis. Results in wt.% for GO and cGO crosslinked with Ca^2+^, Zn^2+^ and Sr^2+^.

The FESEM micrograph with EDS analysis confirmed the presence of the divalent cations of Ca, Zn and Sr after the synthesis procedure with trace amounts (impurities) of chlorine atoms in all the coordination compounds (Fig 2). The EDS data shows that the weight percentage of oxygen atoms in GO and the GO coordinated networks are very similar.

**Fig 3:**
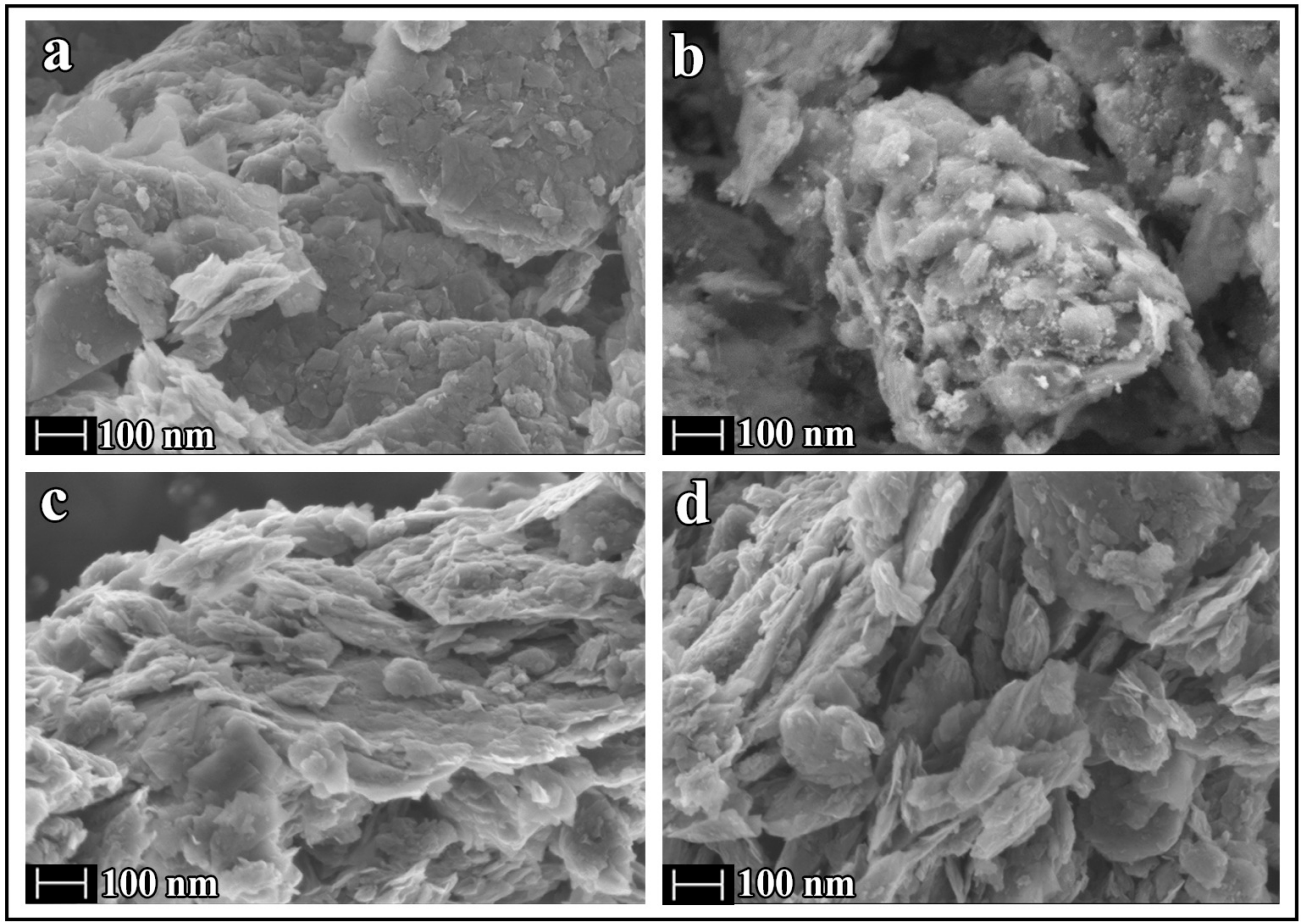
Field emission scanning electron microscopy. Images at a magnification of 50000 of (a) GO and cGO crosslinked with (b) Ca^2+^, (c) Zn^2+^ and (d) Sr^2+^ synthesised at 24°C.

Fig 3A shows that the GO powder used in this study is composed of unfolded GO nanosheets of approximately 100–200 nm in length forming aggregates stacked together mainly on its planar surface by van der Waals forces and π-π interactions. However, GO crosslinked with any divalent cation shows a very different structure of folded GO nanosheets (Fig 3B–3D) linked via divalent atoms. These images show very irregular morphology where the bridging of the GO sheets can be appreciated in the cGO samples in comparison with GO.

**Fig 4:**
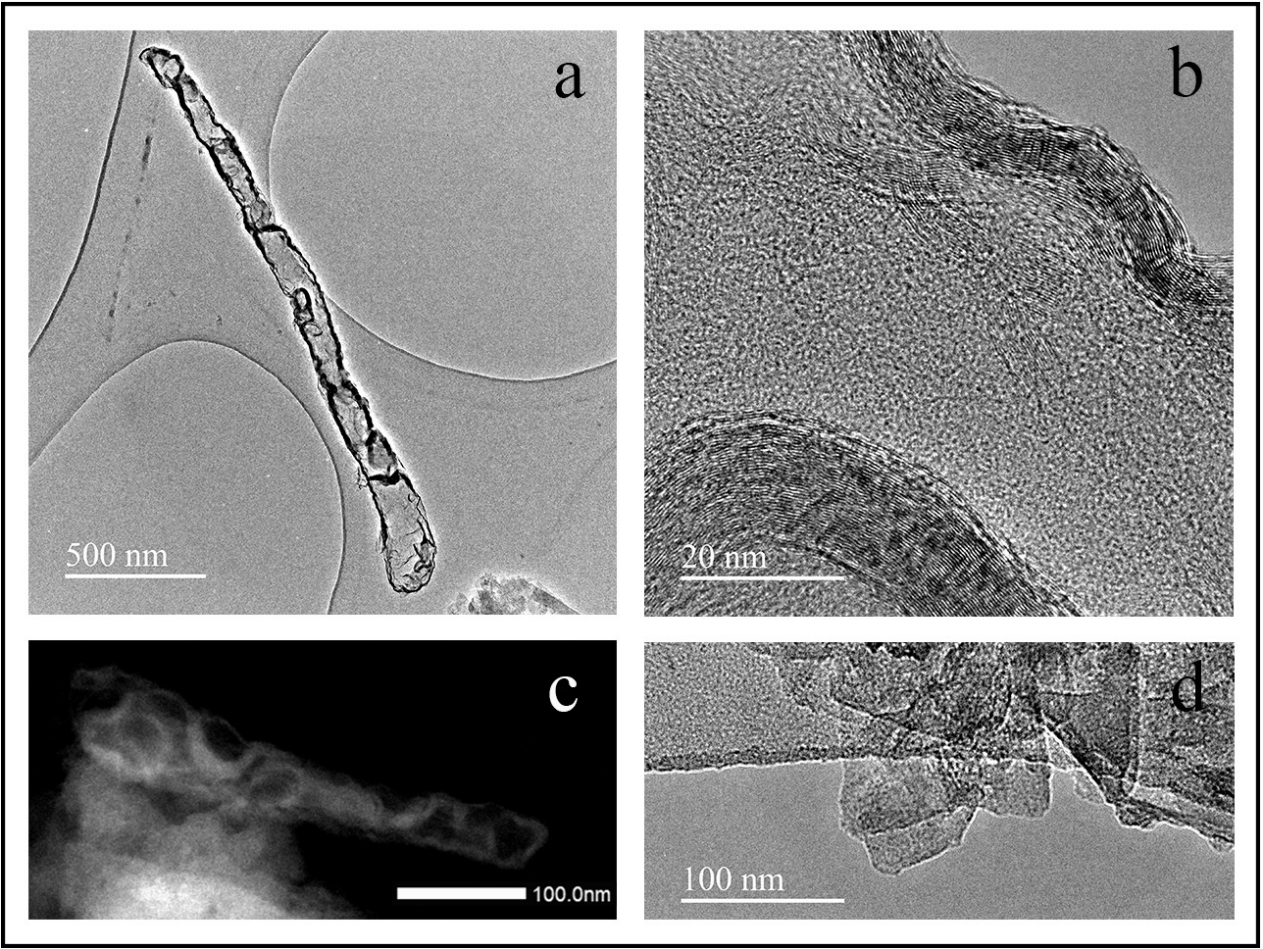
High-resolution transmission electron microscopy. Micrographs at two magnifications (a&b) with STEM dark field (c) of GO crosslinked with Zn^2+^ and uncrosslinked GO nanosheets (d).

When these complex compounds are dispersed in dichloromethane in an ultrasonic bath for ten minutes, irregular micrometer length tubes of continuous crosslinked GO nanosheets can be easily separated and their structure can be very clearly observed by HR-TEM (Fig 4A and 4C). Besides, the lattice atoms of these continuous carbon materials crosslinked with divalent cations of Zn can be directly imaged (Fig 4B).

**Fig 5:**
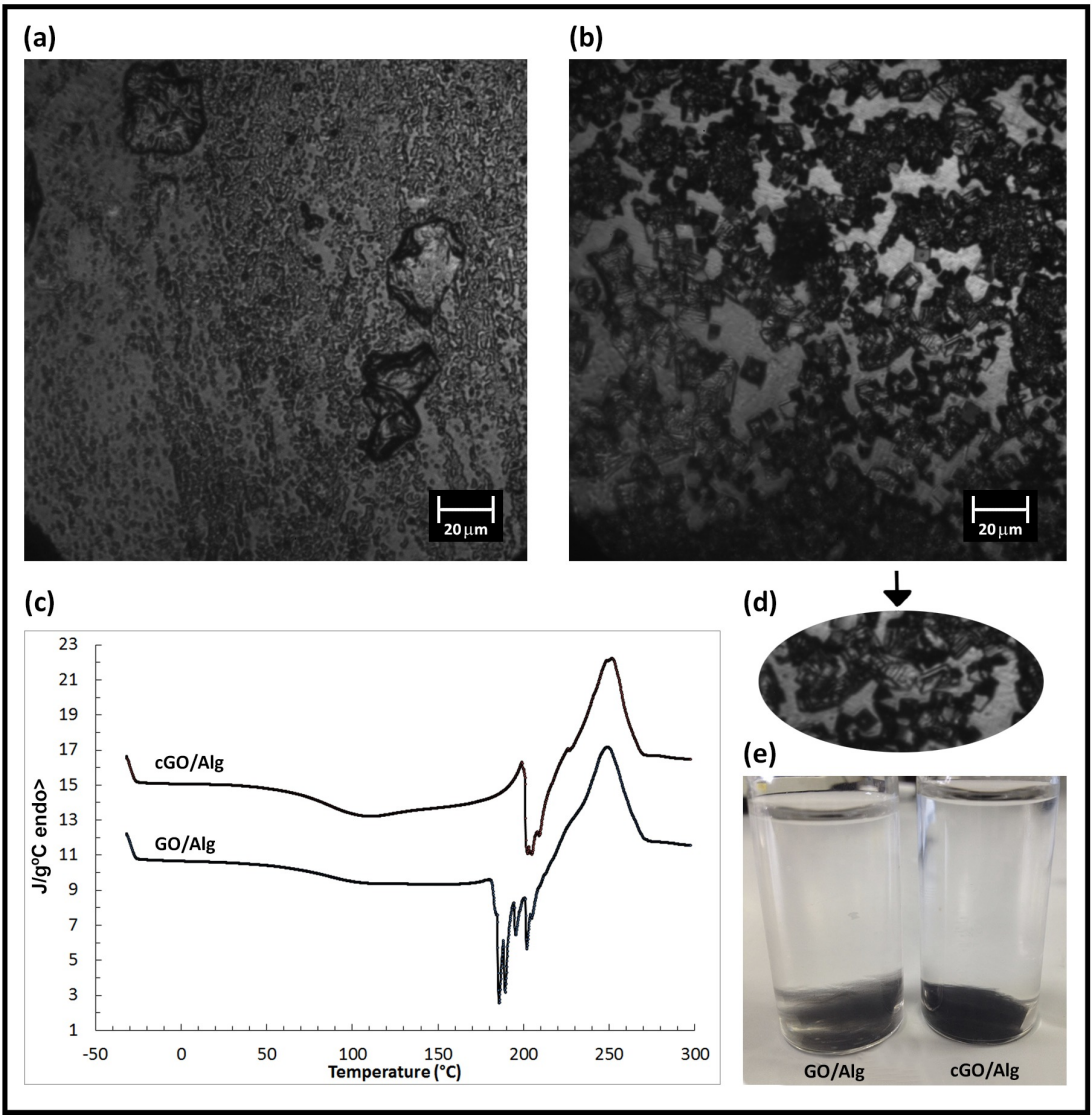
Morphology and differential scanning calorimetry. Confocal microscopy of (a) GO/Alg, (b&d) cGO/Alg, (c) DSC thermograms on heating of these samples at 10°C/min and (e) their morphology after swelling in water for 3 hours.

When crosslinked GO networks are employed to reinforce calcium alginate composites, the morphology of these samples are very different from that of calcium alginate composites prepared with uncrosslinked GO nanosheets (Fig 5). Thus, Fig 5A shows that most GO nanosheets are separated or form small aggregates around the alginate polymer matrix in comparison with the continuous crosslinked GO networks present in cGO/Alg, which can be clearly appreciated (Fig 5B, dark phase) by confocal microscopy in contrast to calcium alginate (light phase).Additionally, continuous irregular tubes of crosslinked GO with divalent calcium atoms can also be visualised in this confocal image forming sometimes right angles between them (see Fig 5D). However, there are three huge irregular micrometer-size continuous GO forms in GO/Alg, which indicate that GO crosslinking also occurs in GO/Alg during the gelation process.

**Fig 6:**
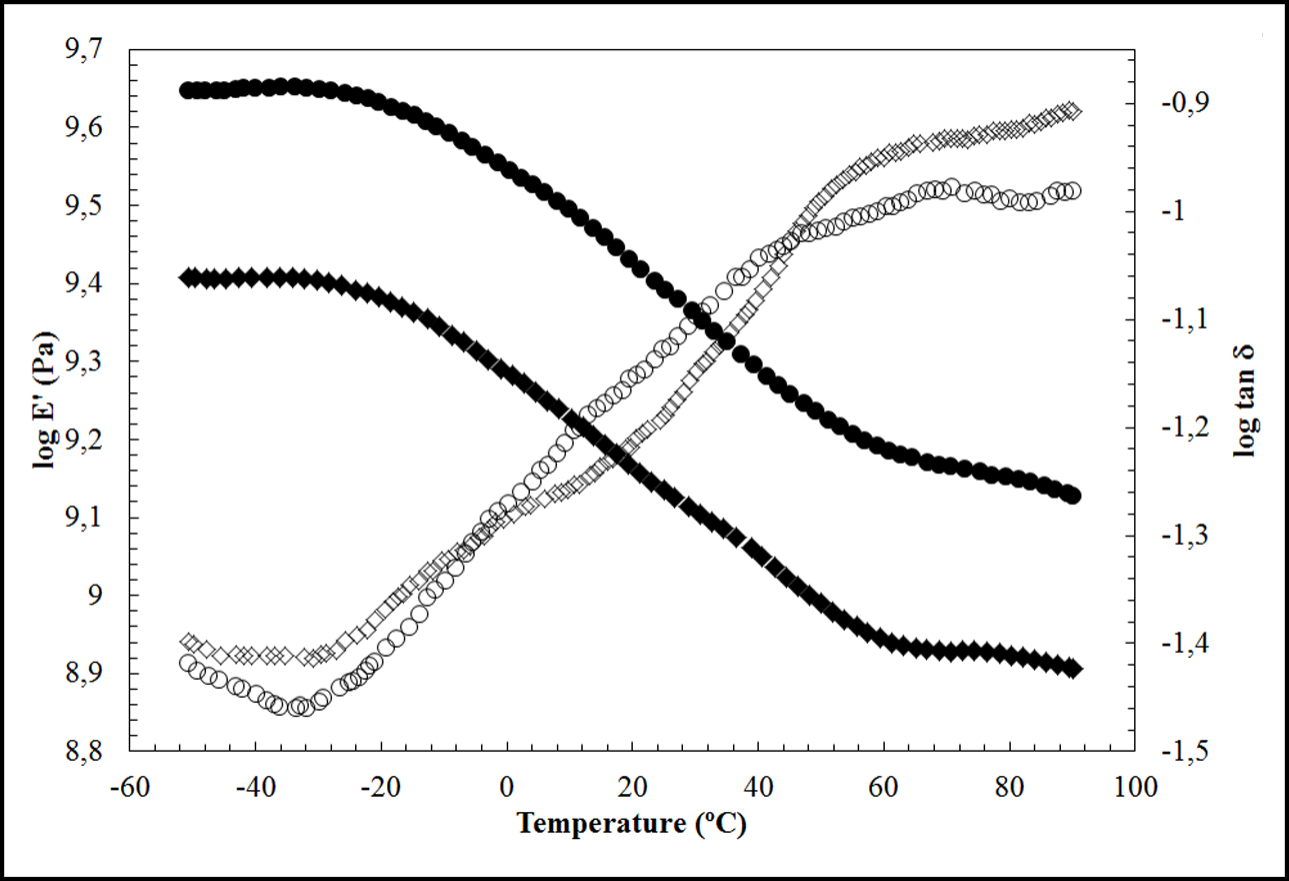
Dynamic mechanical analysis. Temperature dependence of the storage modulus (solid symbols) and the loss tangent (open symbols) of cGO/Alg (◯) and GO/Alg (◇). Only one point out of ten is plotted to obtain a clearer representation.

When the reaction is conducted with cGO, a darker hydrogel is obtained. Similar to graphene, GO is highly transparent in the visible spectrum, the degree of transparency being attributed to electrostatic, chemical, van der Waals interactions and others that, remain largely unknown[59]. However, the transparency of graphene oxide decreases as a function of its number of layers[60]. Therefore, these new alginate composites are much darker because they have folded 3D GO networks instead of uncrosslinked GO sheets. In addition, cGO/Alg is much more consistent as observed by the differential scanning calorimetry (DSC) measurements that show a more homogeneous behaviour with less crystallisation peaks on heating starting at higher temperature (Fig 5C followed by a melting peak related to thermal degradation of the alginate at a higher temperature. Besides, its chemical structure seems to be much better linked after immersion in distilled water for 3 hours at room temperature (24±1°C) (Fig 5E). Hence, bonds between GO nanosheets and alginate chains occur via divalent calcium atoms in both GO/Alg and cGO/Alg. However, continuous crosslinked GO nanosheets linked to the alginate polymer matrix through these bonds produce a higher reinforcement as expected (Fig 6). In this Fig 6, the storage modulus (E′) and loss tangent (tan δ) are investigated by dynamic mechanical analysis (DMA) to determine the performance of these two composite materials under stress at various temperatures. For both samples, a decrease in storage modulus (E’) is observed as the temperature increases because the material undergoes a glass/rubber transition state. It is also noticed that the incorporation of crosslinked GO into the alginate matrix (sample cGO/Alg) leads to a considerable increase in the storage modulus (E’) as compared with the addition of single GO sheets (sample GO/Alg). The raise in the storage modulus is due to the higher reinforcing effect of the irregular graphene oxide tubes and higher interfacial interaction between these tubular carbon materials and alginate that restricts more the mobility of polymer chains at Alginate-cGO interface and high modulus of cGO. The damping factor (tan δ) exhibits a single dynamic mechanical relaxations in cGO/Alg in contrast to the heterogeneous behaviour of GO/Alg with two mechanical relaxations in good agreement with the DSC results.

**Fig 7:**
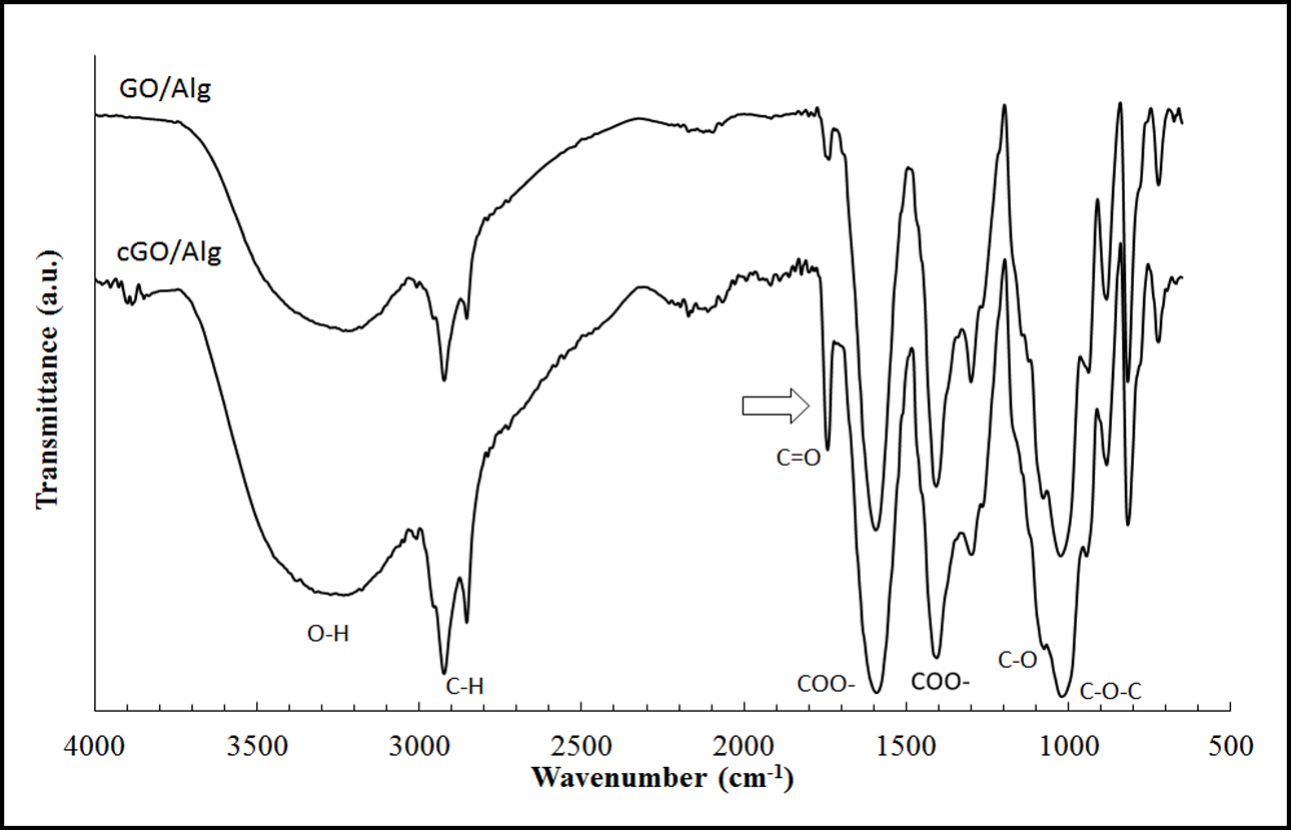
Fourier transform infrared spectroscopy. Spectra of cGO/Alg and GO/Alg from 650 to 4000 cm^-1^.

The FTIR spectra show that the functional groups of GO/Alg and cGO/Alg are very similar as expected (Fig 7). Thus, a broad peak around 3250 cm^−1^ corresponds to OH stretching vibration, the bands at 1600 cm^−1^ and 1414 cm^−1^ correspond to symmetric and asymmetric COO− stretching vibration of carboxylate salt group, and the peak at 1030 cm^−1^ due to the stretching vibration of C−O−C groups respectively[50]. The shoulder peak at 1087 cm^−1^ is attributed to the C−O stretching of sodium alginate[61]. However, the intensity of the peak corresponding to the carbonyl esters (1735–1750 cm−1) increases considerably indicating that this group plays a more important role in cGO/Alg due to the GO crosslinking via divalent cations by coordination chemistry and a slight shift of this peak from 1750 to 1746 cm-1 is observed.

These results support the idea of occurring higher interfacial interaction between tubular carbon materials and alginate in cGO/Alg in good agreement with the DMA and DSC analysis.

In literature, it has been reported that the addition of GO can enhance the storage modulus of alginate-based materials [62,63]. However, to the best of our knowledge, there is not any publication on the enhancement of E’ for composite hydrogels synthesised with irregular graphene oxide tubes with respect to those with single GO sheets. Therefore, these results confirm our hypothesis of these irregular graphene oxide tubes as better reinforcement agents than single GO sheets in alginate. The possibility of including zinc and strontium in the polymer matrix, which impart antibacterial and healing properties, render these new advanced materials very promising in the biomedical field.

## Conclusions

In conclusion, this study reports a new green and low-cost synthetic procedure that has been developed to join GO nanosheets to produce folded 3D crosslinked GO networks by coordination chemistry to form irregular micrometer length tubes. Crosslinking of GO is much more effective using Zn^2+^ and Sr^2+^ at room temperature (24°C) than with Ca^2+^, which requires the temperature to be raised to 34°C to achieve the same crosslinking density. The synthesis of the new calcium alginate composites reported in this study thus show an example of the potential use of these GO networks to reinforce other materials showing a very significant increase in the storage modulus and a more homogeneous structure. Of note, is that this synthetic process is in keeping with the principles of green chemistry and in good agreement with the sustainable non-hazardous synthetic routes with no demands on thermal or sonic energy consumption. These 3D networks of GO coordinated with divalent cations (Ca, Zn, Sr or others) will enable broadening of the field towards the synthesis of new continuous carbon materials and reinforced composites such as alginates with unique electric, thermal, oxidative, and chemical properties with very promising applications in fields of electronics, optics, energy, environmental science and biotechnology in the near future[5,11,64].
